# How to achieve entrepreneurial enterprise performance in entrepreneurial scenario? – Based on the case study of China new energy passenger car company A

**DOI:** 10.3389/fpsyg.2022.946806

**Published:** 2022-12-19

**Authors:** Jianxin Dai, Xiaowei Lu, Huibo Qi, Bo Zhang, Wei Wang

**Affiliations:** ^1^School of Business Administration, Zhejiang Gongshang University, Hangzhou, China; ^2^College of Economics and Management, Zhejiang A&F University, Hangzhou, China; ^3^School of International Studies, Zhejiang Business College, Hangzhou, China

**Keywords:** entrepreneurship, rapid change, enterprise performance, entrepreneurial scenario, new energy

## Abstract

**Introduction:**

With the rapid development of China in recent decades, entrepreneurial scenarios are constantly changing, greatly promoting entrepreneurial practice. The characteristics of China’s entrepreneurial scene, such as institutional differences, economic differences and cultural differences, make it unique. This research is based on a Chinese new energy vehicle start-up. Focus on how to achieve entrepreneurial enterprise performance in this unique entrepreneurial scenario.

**Methods:**

Based on the development process from 2014 to 2021, using entrepreneurial scenario and entrepreneurial performance theory, focusing on the two themes of “what to do” and “how to do”, and adopting exploratory case study methods, the performance of entrepreneurial enterprises was studied.

**Results:**

The study found that in the context of Chinese entrepreneurship, cultural background has the most significant impact on the performance of entrepreneurial enterprises. The accurate prediction of institutional scenarios by entrepreneurial enterprises can improve enterprise performance, while economic scenarios have a negative impact on entrepreneurial enterprise performance.

**Discussion:**

The research shows that in the development process of entrepreneurial enterprises based on China’s entrepreneurial scenario, the governance mode and strategic choice of entrepreneurial enterprises should match the scenarios at different stages. At different stages of development, entrepreneurial enterprises should flexibly adapt to entrepreneurial scenarios and adopt different strategies to reflect their advantages in entrepreneurial scenarios and improve the success rate of entrepreneurship.

## Introduction

In the past 10 years, the global renewable energy economy has increased significantly (Opoku et al., 2020). Many emerging economies have pursued sustainable development policies (Ponce and Khan, 2021), implemented relevant economic policies (Khan et al., 2020a, 2020b), and significantly promoted sustainable economic development. For example, China’s GDP reached 53.9 trillion yuan in 2012, and 114.4 trillion yuan in 2021, with an annual growth rate of more than 6%. While China’s economy is developing rapidly, it has always been implementing policies such as the integration of digitalization and industrialization (Sheng and Dai, 2019), and the Made in China 2025 Power Strategy. China’s entrepreneurial scenario is changing rapidly with this rapidly developing economy. The changing entrepreneurial scenario in China has greatly facilitated entrepreneurial practice and has definitely generated a number of new issues and new trends (Zhou et al., 2020). China’s entrepreneurial scenario has the characteristics of institutional differences, economic differences, cultural differences, etc. In particular, the impact of reform and opening-up on the economic system and the cultural scenario under this economic system model has created the uniqueness of China’s entrepreneurial scenario.

Entrepreneurial scenario affects the entrepreneurial process (Williams and Vorley, 2015), and then affect entrepreneurial performance. Improving entrepreneurial performance is a key task to deepen and consolidate the achievements of reform and development, and also a key problem faced by entrepreneurial enterprises in the Chinese entrepreneurial context. Especially in the past decades of reform and opening-up, a series of changes in e-commerce, mobile Internet, mobile payment and other fields have influenced the change of entrepreneurial scenarios. However, there is still lack of the research on the issues of uniqueness such as how to seize the opportunity to obtain entrepreneurial enterprise performance in different entrepreneurial scenarios, how to deal with scenario differences and changes in the process of entrepreneurship, how to implement different entrepreneurial activities according to the change of scenarios, and how to adapt to rapidly changing scenarios. There have been studies on the interaction between scenarios and entrepreneurial processes, differences in entrepreneurial activities and scarcity of resources in different scenarios (Williams and Vorley, 2015; Bylund and Mccaffrey, 2017; Corbett and Montgomery, 2017). However, there is lack of research on the operating mechanism of entrepreneurial enterprise performance in China’s entrepreneurial context, and there is less research involving rapid changing characteristics in China’s entrepreneurial context, which increases the urgent need for entrepreneurial practical experience and theoretical guidance.

In view of the above consideration and in combination with the entrepreneurial scenario theory, this research is based on a new energy passenger vehicle enterprise as a case study, using the exploratory case method, to investigate the performance of entrepreneurial enterprises in China’s entrepreneurial context, to study and address the limitations and deficiencies under the characteristics of China’s entrepreneurial scenario, and to build a performance paradigm for entrepreneurial enterprises in China’s entrepreneurial context. This paper studies the successful cases of entrepreneurial enterprise performance, especially summarizes and refines the cases in the rapidly changing entrepreneurial context in China, and describes the entrepreneurial enterprise management mechanism, resource allocation mechanism and main measures in its entrepreneurial context to achieve entrepreneurial enterprise performance. This paper only makes a necessary theoretical review and a simple theoretical analysis of entrepreneurial scenarios and entrepreneurial performance theory.

## Literature review

Entrepreneurship is a process that reveals how entrepreneurs create or find entrepreneurial opportunities, and what methods and ways to take advantage of these entrepreneurial opportunities to generate entrepreneurial performance (Shane and Venkataraman, 2000). Entrepreneurship is a way to participate in social and economic activities (Ye et al., 2018). In particular, China is pursuing carbon peaking and carbon neutral policies to achieve green economic growth with less carbon emissions (Khan et al., 2022). This plays an important role in providing more opportunities for entrepreneurship, promoting national economic growth, increasing market volume, enhancing national strength and improving the ecological environment of residents (Nathaniel and Khan, 2020). Different scholars have different understandings on entrepreneurship. Some scholars perceive it as “start-up from scratch” (Lin et al., 2001), while others understand it as “internal entrepreneurship.” The “entrepreneurship” studied in this paper mainly refers to “start-up from scratch,” that is, the process of creating new organizations. Entrepreneurial activities are activities carried out in certain entrepreneurial scenarios, and entrepreneurial scenarios (Shepherd et al., 2019) affect the entrepreneurial process and ultimately entrepreneurial performance.

### Entrepreneurship scenario research

Entrepreneurship scenarios are all factors related to the content, system, culture and economy in the entrepreneurial process, which directly or indirectly affect the entrepreneurial process (Zhou et al., 2020). Entrepreneurial scenario factors have been mentioned by many scholars. At the same time, entrepreneurial scenarios affect and restrict the universality of entrepreneurial research conclusions. Entrepreneurship scenarios are generally divided into three categories: institutional scenario, cultural scenario and economic scenario (Zhou et al., 2020).

Institutional scenario is a recognized rule that must be followed in the process of entrepreneurship. The entrepreneurial process needs to be matched with the laws and regulations representing the institutional scenario (Bylund and Mccaffrey, 2017). The institutional scenario affects entrepreneurial activities (Williams and Vorley, 2015). The dynamic change of the time of the institutional scenario has a great impact on entrepreneurial activities, such as institutional change (Bitektine and Haack, 2015). At the same time, the entrepreneurial process will also have an impact on laws and regulations. The entrepreneurial process can promote institutional change (Shepsle, 1989). There is an interaction mechanism between the entrepreneurial process and institutional scenarios. However, under the specific institutional context of rapid change in China, there is little research on the knowledge reserve and technical capacity requirements necessary for the entrepreneurial process.

Economic scenario refers to the impact of national or regional economic situation on the entrepreneurial process. Macroeconomic scenarios such as macroeconomic freedom (Mcmullen et al., 2008) and macroeconomic crisis have a great impact on the entrepreneurial process. At the same time, microeconomic scenarios such as market maturity (Tan et al., 2009) and market category (Corbett and Montgomery, 2017) will also affect the entrepreneurial process. Economies in transition from planned economy to market economy (Hoskisson et al., 2000).

Cultural scenario is the belief and attitude of relevant people in a region toward particular things. This is an unwritten and decentralized rule that encourages relevant personnel to engage in relevant activities in certain ways (York and Lenox, 2014). The cultural scenario includes three parts: micro level, meso level and macro level. Organizational culture and other elements of thinking that affect management and innovation belong to the micro level (Shirokova et al., 2018). Ethnic culture and religious culture have different impacts on trust judgment and entrepreneurial intensity in the relationship, which is the middle level (Sabah et al., 2014). The level of entrepreneurial orientation and venture capital activities caused by cultural values and norms in different countries is at the macro level (Stephan and Pathak, 2016; Bandera et al., 2018). The majority of scholars have not reached a consensus on such issues of the impact mechanism of cultural scenarios on the entrepreneurial process.

After the reform and opening-up, the institutional, economic and cultural scenarios are constantly changing. China began its reform and opening-up in the late 1970s. Chinese enterprises have experienced the transition from a planned economy to a market economy. The institutional scenario is constantly changing. With the support of sustainable development strategies and policies (Khan et al., 2021), China’s economy has been developing at a high speed for decades. At the same time, with the development of globalization and the global economy, China’s economic scenario is also changing. In the process of dynamic changes in institutional and economic scenarios, cultural scenarios are also changing. China’s unique cultural scenario supports China’s rapid economic development and rapid changes in economic scenarios, and also affects the dynamic changes in China’s institutional scenarios. In this unique and dynamic entrepreneurial scenario in China, many western entrepreneurial theories cannot adapt to the entrepreneurial scenarios in China. We need to constantly understand western entrepreneurial theories and deepen entrepreneurial theories with Chinese characteristics in combination with Chinese entrepreneurial scenarios.

### Entrepreneurial performance

The establishment of entrepreneurial enterprises is not the end point of entrepreneurship. The entrepreneurial enterprises need to constantly improve their ability to resist market fluctuations and risks, improve their corporate immunity, and maintain the long-term development of entrepreneurial enterprises. Enterprise performance has become a problem that every venture needs to face actively. The first performance problem to be solved by entrepreneurial enterprises is survival performance. The survival and growth of entrepreneurial enterprisesis an important performance of the development of entrepreneurial enterprises (Rotger et al., 2012; Degeest et al., 2018). For start-ups, venture capital performance (Li et al., 2014), crowdfunding performance (Anglin et al., 2018) and other capital acquisition performance are the basis for enterprise production and growth. If these performances cannot be guaranteed, the entrepreneurial enterprise cannot survive and develop. On the basis of ensuring the survival of start-ups, start-ups should also pay attention to financial performance such as income and employment growth (Hmieleski and Baron, 2009), sales and profits (Hoogendoorn et al., 2013). These financial performances are the key to the sustainable development of start-ups, and they are also the enterprise performance that needs to be focused on by start-ups. The ultimate goal is to obtain more market shares and excess profits (Monsen and Boss, 2009), and essentially improve their financial sustainability (Mahon, 2013) and legitimacy (Suchman, 1995), and establish positive credibility (Orlitzky and Swanson, 2008) which improves the efficiency based on product market (Rahman et al., 2020). Of course, the performance of entrepreneurial enterprises also includes the improvement of core competence. Startups are likely to engage in for-profit and non-profit activities, which are mainly evaluated by objective measurement standards such as financial performance. This paper mainly studies the profitability of start-up companies, which is mainly reflected in the corporate profitability behavior. Therefore, this paper mainly measures the dimensions of enterprise performance, including enterprise survival and growth, investment and financing performance, sales performance, employment growth performance and technical ability.

### Analysis ideas

**Figure 1:**
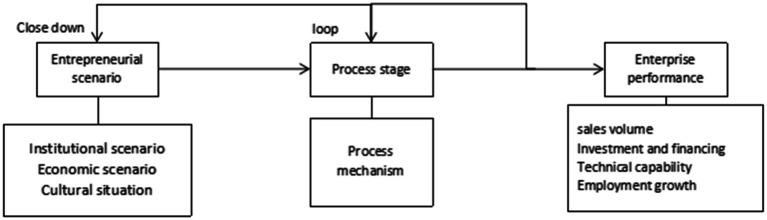
Research framework.

Based on the above literature, this paper believes that the transition from “zero” to “listing” of product R&D of entrepreneurial enterprises is a sign of entrepreneurial performance. With the survival and growth of enterprises in the process of entrepreneurship, entrepreneurial enterprises have developed from micro enterprises to medium-sized enterprises, and finally to large-scale enterprises. In the context of entrepreneurship in China, entrepreneurial enterprises survive and grow in the fierce market competition, improve their comprehensive strength and their ability to withstand the risks of market fluctuation. This paper attempts to open the black box of research on the performance of entrepreneurial enterprises in the context of entrepreneurship in China, and forms a research framework based on the above theoretical analysis, as shown in Figure 1.

## Methodology

### Method selection

The main reasons for choosing the case study method in this paper are: (1) The question studied in this paper is “How to achieve the performance of entrepreneurial enterprises in the entrepreneurial context?” Case study method is appropriate for revealing how entrepreneurial scenarios affect entrepreneurial performance during the development of entrepreneurial enterprises, summarizing the impact mechanism of entrepreneurial scenarios on entrepreneurial performance (Eisenhardt, 1989). (2) There have been relevant studies on entrepreneurial scenarios, but there are relatively few studies on entrepreneurial performance under the existing entrepreneurial scenarios in China. The logical induction between entrepreneurial scenarios and entrepreneurial performance in case study design is used to deconstruct the process mechanism of entrepreneurial performance realization under entrepreneurial scenarios.

### Selection of case enterprises

This paper selects Company A as the case study sample. First of all, there is a certain definition of the age of start-up enterprises. A new energy passenger vehicle company in China was selected, and its establishment time has not exceeded 8 years, which is in line with the time definition of start-up enterprises (Du and Zhang, 2012); Secondly, the enterprise can quickly realize the enterprise performance in the development process, and can analyze the enterprise development process according to the stage; Third, it is typical - Company A already has relevant national qualifications and has a physical production factory; Fourth, the availability of case data - the members of the case study group know many senior executives of Company A, and the members of the case study group work in the same area as Company A, which is geographically close. They have been paying attention to Company A since the early stage of its entrepreneurship, and can obtain first-hand research data by timely understanding the development details and latest strategies of Company A.

### Data collection

The research data are collected from multiple sources through semi-structured interviews, field observations and second-hand data to achieve “triangular verification” of the research data (Yin, 2017) and ensure the reliability and validity of the research data in this paper.

#### Semi structured interview

The research team used purposive sampling (Luo et al., 2015), and interviewed the most valuable information, mainly those with higher administrative level or core project management. Finally, three senior managers were selected, all of whom were co-founders of the company and seven middle-level managers. They joined the company when the company was founded, implemented the company’s decision-making strategy, understood the strategic intent, and were particularly familiar with the company’s development process and development details. These interviewees, who are friends or introduced by friends with the relevant personnel of the case group, are familiar with each other and can honestly exchange details in the development process of the entrepreneurial enterprise. Even some of the opinions carried out by the managers were partially optimized for the implementation in the company after listening to the suggestions of the interviewers.

Due to the busy work schedule of the interviewees, in order to facilitate the interviewees to prepare before the interview, it is necessary to make an appointment for the interview time in advance, and send the interview outline to the interviewees in the form of WeChat or email ahead of schedule. After communication beforehand and the consent of the interviewee, the interview can be conducted face-to-face.

Before the interview, the purpose of the interview information was explained and a confidentiality agreement was signed. After the interview, the transcripts shall be sorted out immediately, which shall be converted into research-worthy information after checking with the interviewees, and the records of the interview process shall be deleted according to the confidentiality agreement.

The interview lasted for 5 years from January 2016 to May 2021, with a cumulative interview time of about 1,220 min, and approximately 130,000 words of translated texts, as shown in Table 1.

**Table 1:** Semi structured interview information.

Serial number	Interview time	Object	Position	Number of interviews	Interview duration (minutes)	Number of words transcribed
1	2017.3	AGM	Vice general manager	1	0	7,807
2	2018.5–2019.5	BGM	Vice general manager	2	0	9,454
3	2016.3	CGM	Vice general manager	1	0	4,139
4	2016.1–2018.12	AM	Minister	8	0	46,632
5	2016.1–2018.3	BM	Minister	5	0	22,056
6	2016.1–2018.2	CM	Minister	3	0	14,192
7	2017.8–2019.1	DM	Minister	2	0	8,647
8	2017.8–2018.3	EM	Minister	2	0	10,923
9	2018.6	FM	Minister	1	0	3,591
10	2019.1	KM	Minister	1	0	3,464

#### Field investigation

The research team collected the company’s development information, personnel information and unclassified fund information through friendship, and was lucky to be invited to participate in some non business secret meetings to learn about some of the company’s operations (unclassified). We visited and learned about project construction progress, product research and development progress and other process information during the entrepreneurial process, and collected about 50,000 words of records.

#### Second hand data

Search the website for text and picture information about enterprise development, product R&D and factory construction progress since the establishment of the enterprise. The information obtained from second-hand materials positively verified the accuracy of the information obtained through semi-structured interviews and field visits.

### Variable measurement

In the formation process of key dimensions, this paper refers to the research results (Sassmannshausen and Volkmann, 2018) and arranges the measurement methods suitable for this study.

#### Measurement of entrepreneurial scenarios

For institutional scenarios, this paper determines the technical environment and policy factors as the measurement dimensions. The technological environment, especially new technological breakthroughs, creates entrepreneurial opportunities. Policy factors, especially incentive policies, can provide entrepreneurial opportunities for entrepreneurial enterprises.

For economic scenarios, this paper takes market environment, technical equipment, material resources and capital resources as measurement dimensions. The changing market demand provides opportunities for entrepreneurial enterprises. Technical equipment is necessary for start-up enterprises in the process of entrepreneurship; Material resources are the material resources needed by start-ups to produce or verify products in the process of entrepreneurship; Capital resources are the capital resources needed by start-ups in all aspects of operation.

Cultural scenarios are divided into normative conventions and behavioral conventions as measurement elements. Normative practices mainly include organizational concepts, awareness and cognition. Behavioral conventions refer to specific behaviors that are finally taken based on the guidance of normative conventions through the process of learning and trial and error (Luo et al., 2015).

#### Performance measurement of start–up enterprises

The performance of entrepreneurial enterprises is a holistic concept that can reflect the final results of entrepreneurial activities and measure the extent to which entrepreneurial organizations achieve their goals. It is a key indicator to measure the success and process of entrepreneurship (Tian and Ding, 2017). Researchers mainly measure enterprise performance from the perspective of new products and new technologies (Lai et al., 2013). Based on the research ideas of existing scholars, this paper takes the survival and growth of enterprises, investment and financing, sales, employment growth and technological capabilities as the performance of entrepreneurial enterprises. Among them, the technical ability is mainly measured by comparing the industry level.

### Coding

#### Multi level coding process

This paper encodes the case data according to the data content and data source (see Table 2 for the source coding rules).

**Table 2:** Coding source rules.

Data sources	Data classification	Source code
Semi structured interview	Interview the deputy general manager to obtain information	AGM
	Interview the deputy general manager to obtain information	BGM
	Interview the deputy general manager to obtain information	CGM
	Interview the technology minister to obtain information	AM
	Interview the r & d director to obtain information	BM
	Interview the personnel director to obtain information	CM
	Interview the production director to obtain information	DM
	Interview the quality director to obtain information	EM
	Interview the purchasing director to obtain information	FM
	Interview the sales director to obtain information	GM
On-the-spot investigation	Obtain data through on-site investigation	FS
Second hand information	Through website	TW
	Publicity materials	PM
	Annual report and internal reporting materials	AR

With the help of QSR NVivo (version No.: 10.0.573.0 SP5 (64 bit)) software, the case data is encoded at the first level according to the division stage of the case. The results show that there are 45 first-class items in stage 1, 53 first-class items in stage 2, 52 first-class items in stage 3 and 55 first-class items in stage 4. Then, according to the 4 dimensions and 10 measurement dimensions, the primary items are allocated to 11 secondary items. Among the 4 primary items, the keywords of dimensions and measurement variables are shown in Table 3.

**Table 3:** Coding statistics of dimensions, measurement variables and keywords.

Construct	Variable or dimension	Keyword	Development stage				Subtotal
			Stage1	Stage2	Stage3	Stage4	
Institutional scenario	Technical environment	The technology gap is not large; improve the ability level; requirements for improvement; improve the level; advanced technique	4	5	4	4	17
	Policy factors	Encourage entrepreneurship; encourage the development of new energy; subsidies decreased; stricter production license; subsidy reduction	5	6	5	5	21
Economic scenario	Market environment	High market growth rate; large demand for products; foreign capital entry; fierce competition; customer requirements improved	8	9	9	12	38
	Technical equipment	Limited resources; active contact; active contact; active communication; free assistance	4	5	5	5	19
	Material resources	Insufficient materials; improve the ability of manufacturers; stable improvement; actively seek cooperation	4	5	5	6	20
	Financial resources	Insufficient funds; select contact; abundant funds; invest in other projects	3	4	4	5	16
Cultural scene	Normative practice	The organization does not limit the scope of work; gradually improve the work responsibilities; process oriented; refinement of department responsibilities	5	4	5	6	20
	Behavioral conventions	Consult experienced personnel; work according to the process; act according to their duties; cross departmental communication is difficult	6	7	7	6	26
Development performance	Sales volume	No sales; sales exceeded 0; sales increased significantly	1	1	3	4	9
	Product development	No products; product offline; mass production of products; trial production of new products	2	3	3	2	10
	Investment and financing	Introduce external investment to meet the capital needs of enterprise development	1	2	2	5	10
	Technical capability	General technical ability; problem solving; leading in the industry; problem solving ahead	4	5	5	4	18
		Total	47	56	57	64	224

#### Multi level coding strategy

In order to ensure the reliability of this study, the case study team has been continuously verifying and confirming from the time of case data collection and transformation. For the converted interview text, the case study team sends it to the interviewees for confirmation in time, and modifies it according to the feedback. In the process of data coding, two researchers completed the coding independently, and then confirmed it. If the results are consistent, they proceed with the analysis. If the results are inconsistent, they look for the differences and reach an agreement after discussion and confirmation.

### Phase division

The existing scholars have rich research methods on the stage analysis of enterprises. From the perspective of stage analysis, analyzing the development and evolution logic of enterprises in chronological order will help to further explore the specific characteristics of each stage (Jiang et al., 2006). Therefore, this paper refers to the development stages divided by Lewis and Churchill (1983) and other scholars. Combined with the dynamic matching process of entrepreneurial scenarios in the development process of Company A, the specific characteristics of each stage in the development process of the case enterprise are divided into four development stages, as shown in Table 4.

**Table 4:** Division of entrepreneurial process stages of company A.

Stage	Stage1	Stage2	Stage3	Stage4
Enterprise status	Start up	The product is successfully offline	Mass production sales	Leading sales
Time frame	2 october 2014 to January 2016	February 2016 to October 2017	October 2017 to October 2018	November 2018 to present
Enterprise performance	Skill accumulation stage, no product, no sales	Skill improvement, formal product offline, no sales	The skills were further improved, the formal products were mass produced, and the sales exceeded 0	Skill improvement, product listing and sales promotion

## Case studies

**Figure 2:**
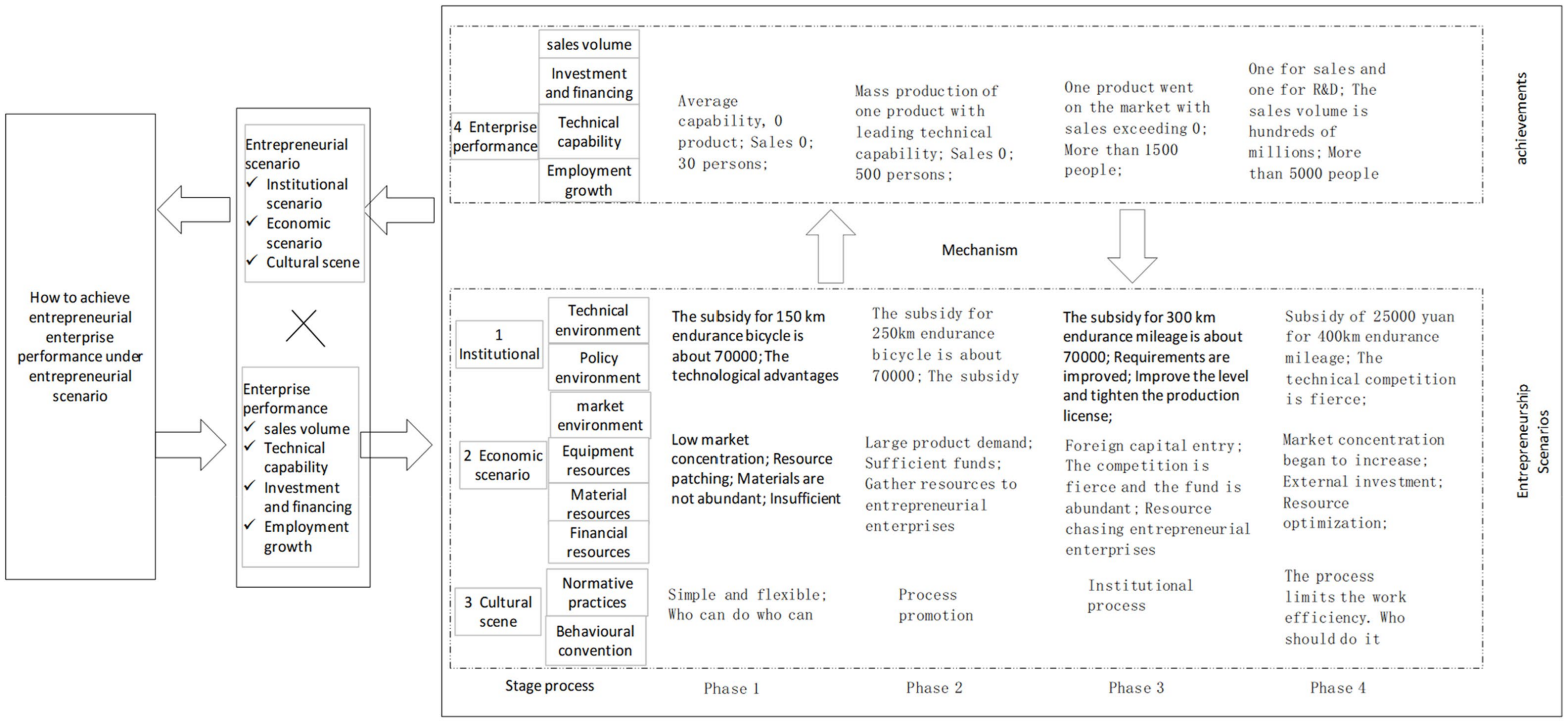
Analysis process of the case company entrepreneurship.

The state adopts a policy of changing subsidy policies for the development of new energy vehicles to stimulate investors and entrepreneurs to promote the development of new energy vehicle industry. Company A is a new energy passenger vehicle enterprise established in line with the development trend. In the process of development, policy subsidies continued to decline, and the pressure on enterprise development continued to increase. The enterprise can grasp the entrepreneurial situation, develop rapidly along the established strategic objectives, and achieve entrepreneurial enterprise performance. The process framework of case enterprise analysis is shown in Figure 2.

### Phase 1

The cross enterprise mature team accelerates the development and maturity of the start-up enterprise and shortens the development cycle of the enterprise. The entrepreneurial team’s grasp and perception of the entrepreneurial situation and confidence in the development trend of the new energy industry constantly stimulate the entrepreneurial team (Bing et al., 2015). After deep thinking, the core members of the entrepreneurial team left the original company in 2014, resigned under the policy incentives at that time, and used the team members’ contacts to integrate and transform knowledge (Kotabe et al., 2011) to pool resources to create a new enterprise. Entrepreneurial team members are mainly people or subordinates who are familiar with the “circle” of new energy passenger vehicles. The entrepreneurial team is built on the basis of personal intimacy, has close personnel relations and high emotional recognition, (Francis and Sandberg, 2000) can actively cooperate and assist, and has efficient and rapid information communication. Get familiar with each other’s comprehensive ability level, solve problems and formulate implementation plans through discussion and negotiation. In principle, “who can do what he can,” there is no strict process management assessment system, such as management evaluation, which is mainly determined by the rule of man (Eisenhardt, 2013).

The entrepreneurial team has clear objectives and unified ideas, and overcomes the difficult period of lack of funds in the early stage of establishment. At the beginning of the establishment of Company A, it experienced a difficult period of 5 months. There was not enough funds to pay wages. The only funds were used for product R&D, which did not affect the progress of product R&D. During this difficult economic period, the team has a unified idea and consistent goals, and many employees even borrowed money to make a living and continue to work in the company. The whole team showed strong cohesion and sense of goal and worked hard to realize their dreams. In the second half of 2015, an external investment was introduced to meet the company’s development capital needs and successfully overcome the company’s early development difficulties.

In the phase 1 of overall analysis, the institutional scenario stimulates the development of enterprise product R&D and technical capability, while the economic scenario inhibits the development of enterprise product R&D and technical capability, and the cultural scenario ensures the survival and growth of start-ups. The survival and growth performance of the enterprise was remarkable, and the introduction of external financing avoided the failure of the enterprise in time, but the financial performance such as sales was not significant. See Table 5 for typical data and coding analysis results.

**Table 5:** Cited examples and coding results at the phase 1.

Construct	Variable or dimension	Typical data	Source	Keyword	Coding results
Institutional scenario	Technical environment	There was little difference in technical level among enterprises, and the ability level was basically the same	AGM	The technology gap is not big	Technical advantages are not obvious
	Policy factors	National policies helped the development of this field, especially in the first tier cities with large demand. National policies further promoted the demand for products	BGM; CGM	Encourage entrepreneurship; Encourage the development of new energy	Policy incentives
Economic scenario	Market environment	The market showed a trend of rapid development, and there was a strong demand for products. There was no brand effect in the market, and consumers did not form a consistent brand tendency	KM	High market growth rate	Low market concentration
	Equipment resources	The resources of equipment were very limited. To meet our ability requirements, we should also make payment terms as good as possible, otherwise too harsh conditions would put too much pressure on our funds	AM; FM	Limited resources	Resource patchwork
	Material resources	When choosing resources, large enterprises are not interested in us at all, and small enterprises cannot meet our ability requirements, so it is very tangled to find material resources	FM	Insufficient materials	Resource patchwork
	Financial resources	At the beginning of entrepreneurship, the funds in the account were insufficient. They were busy with R & D expenses and everyone’s wages	CGM	Insufficient funds	High financial pressure
Cultural scene	Normative practice	In the process of work, the organization was generally limited by the division of work, and there was no strict limit on who did the work and who was responsible for those work	CM; AM	The organization does not limit the scope of work	Simple and flexible
	Behavioral conventions	According to our experience, we were familiar with each other. Whoever has rich experience will find more experienced people to ask for advice in the process of work and check the problems	CM; BM	Consult experienced personnel	
Enterprise performance	Sales volume	There was no sales revenue at the beginning, and it was basically put into the R & D stage	AGM; KM	No sales	Sales revenue 0
	Product development	Product R & D in these start-ups, the R & D progress was similar to that of other enterprises, which are in the R & D stage	BM; FS; PM	No products	Industry equivalence
	Investment and Financing	Introduced external investment to solve the capital problem in the early stage of entrepreneurship	CGM	Introduction of investment	About 100 million
	Technical capability	Everyone’s technical ability level is relatively high. Backbone personnel generally have about 10 years of work experience, and the team ability is in running in	AM; DM	Average technical ability	Industry equivalence

### Phase 2

After several years of development, Company A has formed a strong entrepreneurial atmosphere within the enterprise and continuously achieved phased results. In mid-2016, the trial production products were rolled off the production line, which proved the company’s technical ability and product R&D strength; Formal tooling products were rolled off the production line in 2017; in the same year, it obtained the qualification of national ministries and commissions, and solved the problem between life and death. In 2017, the construction of the company’s factory area was completed, with independent production capacity. The new office area was put into use, and the image was more positive.

With the continuous increase of work and business, the scale of the company has further expanded, with a total number of more than 500 people. Due to the increase of business demand, the division of labor was gradually refined, but the lack of company process led to work errors. For example, in the process of product trial production, the detailed planning of work was obviously not comprehensive and the system process was missing, which affected the quality of verification work. All departments took the initiative to carry out work, made up for the impact caused by the lack of work flow through efficient and fast information communication, actively improved the system and process. The overall relationship is harmonious and the problems were solved quickly. The team has gradually realized the advantages of process control, which was that the overall situation of the organization should be streamlined and highly efficient. In terms of capital, with the large-scale development of the company, there was a shortage in capital, and a larger-scale capital investment was needed.

Throughout the whole phase 2, cultural scenarios promoted the improvement of enterprise performance, such as product R&D and technical capability development. Economic scenario has insignificant effects on product R&D and technical capability development. Overall, the enterprise has achieved mass production of new products and the enterprise performance such as its technical capacity has been significantly improved. See Table 6 for typical data and coding results.

**Table 6:** Citation examples and coding results in phase 2.

Construct	Variable or dimension	Typical data	Source	Keyword	Coding results
Institutional scenario	Technical environment	The products of powerful enterprises have been maturing, and the technical capabilities of power batteries, motors and electronic controls have been continuously improved	BM; BGM	Ability level improvement	Improvement of technical capability requirements
	Policy factors	Policy subsidies began to decline, but they still encouraged the development of the industry	CGM; AM	Subsidy decline	Preferential policies began to decrease
Economic scenario	Market environment	Market competition began to appear, and the market demand and sales volume showed double-digit growth	KM; BM	Large demand for products	Low market concentration
	Equipment resources	Equipment resources began to be abundant. Some equipment manufacturers took the initiative to find us and give us a trial. There could be many preferential payment terms	FM; BGM	Active contact	Resource aggregation
	Material resources	Capable manufacturers began to cooperate with us, and the continuous improvement of material resources and quality is very important to the stability of our product performance	DM; EM; BM	Improvement of manufacturer’s ability	Resource aggregation
	Financial resources	Large investment institutions began to pay attention to us and conduct continuous investigation. We could make selective contact among potential partners. We would not contact some institutions with ordinary strength	CGM; BGM	Select contact	Reduced financial pressure
Cultural scene	Normative practice	The lack of responsibilities led to the reduction of internal communication efficiency and the omission of work. Working according to the responsibilities could reduce the risk of omissions.	EM	Work responsibilities are gradually improved	Gradual normalization
	Behavioral conventions	In the working process, the work was carried out step by step with reference to the work flow, which improved the efficiency and reduces omission of work	DM	Work according to the process	
Enterprise performance	Sales volume	There was no sales revenue, but the supplier network in key cities has been completed	DM; CGM; KM	No sales	Sales revenue 0
	Product development	The trial products are rolled off the production line and met the preliminary product requirements. The formal products are rolled off the production line, and the performance reached the industry level	BM; EM; FS; PM	Product offline	Industry leading
	Investment and financing	The company introduced external investment to solve the problem of the lack of enterprise development funds	CGM	Introduction of investment	About 1.2 billion
	Technical capability	Many problems have been found when the trial products were rolled off the production line, which could be solved quickly, and many problems could be solved intensively before the formal products were rolled off the production line	AM; EM; AR	Problem solving	Industry equivalence

### Phase 3

External capital investment stimulated the rapid changes in enterprise micro-cultural scenarios. The entrepreneurial team did not understand the operation mode of the capital market and the control rights of the company in the process of introducing external investment. When a large amount of funds came in, the entrepreneurial team lost control of the company and only had the right to operate. The intervention of new shareholders has brought new ideas, new working methods, new knowledge structure and new values, which not only complemented the deficiencies found in the development process of the company, but also led to the conflict between the new working methods and the original working methods of the entrepreneurial team. Thus the team cohesion was in urgent need of running-in. The company’s organization has been continuously refined and split. The number of organizations has increased. A large number of new employees have entered. The staff number has surged by 1,500. Moreover, the salary gap has increasingly widened and the sense of fairness among personnel has gradually lost, which led to the decreasing work enthusiasm. The overall working atmosphere has changed. In this stage, though lack of entrepreneurial mentality among the new employees, the awareness of professional tasks was common. The work atmosphere based on responsibility was accepted by everyone, and the awareness based on completing index tasks gradually became dominant, while the awareness based accomplishing goals and overcoming difficulties was gradually weakened. In addition, cross-departmental cooperation was gradually reduced, and personnel relations are increasingly alienated. The main task of middle managers is to ensure the effective transmission of information from senior managers to operation managers (Floyd and Lane, 2000). Middle managers play an important role in the information input, processing and sharing in the organization (Liu and Xi, 2021).

The decline in policy subsidies has forced product R&D to optimize and further increase the cruising range, so as to meet the expectation of policy subsidies in the future. In terms of the company’s performance, it obtained the production license from national ministries and commissions in 2018, achieved the strategic goal of the entrepreneurial team, and successfully put the products on the market. The sales volume in that month exceeded the expectation, and the first batch of funds was returned. The company’s system and process were further revised and improved, the division of responsibilities of various departments was gradually clarified, and the prototype of “rule of law” was gradually established. However, due to the fact that the organization was huge, and the efficiency and quality of system communication were gradually reduced. The enterprise decision-making mode was constantly changing, forming a decision-making in the form of report, and the implementation was carried out according to the decision-making results. Increased rigor and intensification of reporting processes at all levels of the company resulted in reduced efficiency.

Summarizing the phase 3, it shows that the institutional scenario encourages the improvement of enterprise product R&D and technical capability, while the gradual change of economic scenario inhibits enterprise performance, and the impact mechanism of cultural scenario on enterprise performance is not significant. In this stage, product R&D continued to develop under the comprehensive effect, and technical capability remained in the lead, the sales achieved a breakthrough of 0, and enterprise performance was significant. See Table 7 for typical data and coding results.

**Table 7:** Cited examples and coding results in phase 3.

Construct	Variable or dimension	Typical data	Source	Keyword	Coding results
Institutional scenario	Technical environment	With the continuous increase of the number of products in the market, the requirements for product technical ability were continuously improved, and the technical ability level of relevant products was continuously improved.	AGM; BM; EM	Requirements for improvement; raise to higher level	Increased technical threshold
	Policy factors	Policy subsidies were greatly reduced, and the requirements for product costs were higher and higher. According to the early product research and development, the listing would lose money. The production license was radually tightened.	CGM; KM; BM	Stricter production license	Increased entry threshold
Economic scenario	Market environment	With the growth of market demand and the entry of international brands and the determination of factories of foreign-funded enterprises, the competition in the market environment began to be fierce.	FS; FM; KM; BGM	Foreign capital entry; fierce competition	The competition began to be fierce
	Equipment resources	International enterprises began to contact and provide equipment resources.	FM; AM	Active contact	Resource pursuit
	Material resources	Large enterprises began to provide materials. The stability of material supply was improved and the quality level was further improved.	FM; AM; EM	Stable improvement	Resource pursuit
	Financial resources	With the strong support of major shareholders and the injection of large amounts of funds, the company’s capital problems have been solved and the company was no longer worried about capital problems.	CGM	Abundant funds	Abundant funds
Cultural scene	Normative practice	The division of responsibilities has been continuously refined, the work efficiency in the work process has been constantly improved, and the work omissions has been reduced; The workflow has been continuously refined and improved.	CM; AGM	Process oriented	Organization organization
	Behavioral conventions	Work according to the process and quickly solve the work faced	DM; EM; BGM	Act according to your duties	
Enterprise performance	Sales volume	The first car was launched and the sales momentum was strong	DM; CGM; KM	Have sales	Sales of nearly 100 million
	Product development	After obtaining the national production license, the official products began to batch, and the research and development of the next product began	FS; PM	Mass production of products	Domestic equivalent
	Investment and financing	Introduced external investment to solve the problem of funds for the rapid development of enterprises	CGM	Introduction of investment	About 2 billion
	Technical capability	The overall technical capability has been continuously improved. The overall level of intelligence has been improved, and the level of product R & D was leading in the industry	AM; BGM	Leading in the industry	Industry leading

### Phase 4

After losing the company’s management and decision-making power, the entrepreneurial team regained control of the company in 2019 through several rounds of efforts. At present, the company has more than 5,000 employees, complete systems and processes, and basically realizes the “rule of law” state of the company’s management. The reporting and decision-making mechanism is used for business decision-making. The operation process of the enterprise is long, and the work efficiency is gradually reduced together with the cooperation efficiency between departments as well as the information communication efficiency. The personnel of each department communicate on the premise of their respective responsibilities, and the team relationship is linked based on their responsibilities. Most members take completing the work as their work goal in order to expect better work performance. Performance pressure urges managers and employees to work hard to achieve performance (Gardner, 2012). At present, two products have been successfully listed, the monthly sales volume has exceeded the sales target of 10,000 units and the annual sales volume has exceeded 10 billion yuan. The external influence of the company has been greatly improved, and the brand construction has been steadily strengthened. At the same time, more research and development of new products are carried out.

Entrepreneurial enterprises have moved from the phase 1 to the phase 4 of mass production. Economic scenarios stimulate the development of enterprise product R&D and technical capabilities, while cultural scenarios inhibit the development of enterprise product R&D and technical capabilities. Under the comprehensive effect, the enterprise’s product R&D and technical capabilities have developed significantly, the sales have reached hundreds of millions, and the enterprise performance has been significantly improved. See Table 8 for typical enterprise data and coding results.

**Table 8:** Citation examples and coding results in phase 4.

Construct	Variable or dimension	Typical data	Source	Keyword	Coding results
Institutional scenario	Technical environment	Intelligent driving and other advanced technologies continue to appear, and advanced technologies continue to be tested and verified.	BM; AGM; BGM	Advanced technique	Fierce technical competition
	Policy factors	Policy subsidies have entered the post subsidy era, but the policy support for the industry has not changed and has always been the main direction of development.	BGM; CGM	Subsidy reduction	Serious decline in subsidies
Economic scenario	Market environment	The market demand is still relatively large, but the market’s requirements for products continue to improve. Products with general quality and style are no longer popular, and the market begins to be picky.	FS; AGM; KM; AR	Customer requirements improved	Market concentration began to increase
	Equipment resources	Enterprises with strong ability in the industry continue to conduct technical exchanges with the company and are willing to cooperate and provide technical support.	AM; AM; FM; BGM	Active communication; Free assistance	Resource optimization
	Material resources	The suppliers who cannot provide qualified logistics on time shall be subject to formal assessment, and the suppliers who cannot meet the requirements shall be disqualified.	AM; AM; FM; KM	Actively seek cooperation	Resource optimization
	Financial resources	There are abundant capital resources, and there are surplus funds to invest in other projects. At present, other projects are being carried out in a tense and orderly manner.	CGM; BM	Invest in other projects	Abundant funds
Cultural scene	Normative practice	The responsibilities of the Department shall be gradually refined, and the implementation of the responsibilities without shall be prohibited, so as to reduce the work and reduce the loss; The division of work is gradually refined, and the number of departments is increasing. We recognize the importance of work refinement.	BM; AGM; BGM; CM; EM	Refinement of department responsibilities	Internalization orientation
	Behavioral conventions	In the process of work, no assistance will be given to the work that does not involve responsibility, and no active responsibility will be taken for the work that does not actively require. According to the detailed division of work, the cross-departmental work involved in the process of work is increasing.	EM; BM; DM	Cross departmental communication is difficult	
Enterprise performance	Sales volume	The goods are highly valued in the market and have a large sales volume. All departments of the company are very nervous.	DM; CGM; KM; FM	Large sales	Hundreds of millions of sales
	Product development	Products are on the market, 2 products are on the market, and 2 products are under development.	FS; PM	Trial production of new products	Domestic leading
	Investment and financing	External investments are introduced to solve the problem of enterprise operating capital.	CGM	Introduction of investment	About 12 billion
	Technical capability	The team has strong overall technical ability and can identify problems in advance, prevent and solve them. The team is able to solve all aspects of products, plan products beforehand and reduce problem.	AR; EM; BM; AM	Problem solving ahead	Industry leading

### Case summary

Through the in-depth analysis of the entrepreneurial process, this paper finds that: ① in the phase 1, not only is the threshold low in the institutional scenario, but also the market concentration is low in the economic scenario, and the resources are scarce. In terms of cultural scenario, the team mainly inherits the original behavioral practices; enterprise performance is not significant. ② in the stage of phase 2, the threshold of institutional scenario is gradually raised. In terms of economic scenario, resources begin to gather actively toward the start-ups. In the aspect of cultural scenario, the normative practices of the team are steadily implemented, and process control emerges. Enterprise performance is significantly improved. ③ in the phase 3, the threshold of the system scenario is further raised, the market concentration in the economic scenario begins to intensify, the competition becomes fierce, and the resources start to gather actively. In terms of cultural scenario, normative practices and behavioral practices have been strengthened at the same time. Enterprise performance achieves breakthroughs; ④ in the phase 4, the threshold of institutional scenario is raised; in the aspect of resources in the economic scenario, external resources actively invest costs and select high-quality resources to support the development of enterprises. In terms of cultural scenario, normative practices are strengthened and affect behavioral practices. In addition, enterprise performance improves steadily.

## Results and discussion

China is carrying out new reforms (Zhou et al., 2020), which will inevitably create a lot of opportunities and generate a lot of research topics. This study focuses on China’s entrepreneurial context, aiming to expand the relationship between entrepreneurial context and enterprise performance through case studies, and provide empirical breakthroughs. Changes in the institutional, economic and cultural scenarios in the past 7 years have a significant impact on the performance of Enterprise A.

### Cultural scenarios have the most significant impact on entrepreneurial enterprise performance

The cultural context adjusts the internal organizational environment and promotes all personnel to integrate into the entrepreneurial plan (Covin and Slevin, 2002), which helps to improve the performance of entrepreneurial enterprises. In the early stage of difficult entrepreneurship, although the case company did not have enough funds to pay salaries, it still survived (Rotger et al., 2012). The only funds were used for product research and development, which was still going on smoothly, and the company continued to grow (Degeest et al., 2018). In this period of financial difficulties, the team had a unified idea and consistent goals, and the whole team showed strong cohesion and entrepreneurial intent (Douglas, 2013). The main members of the entrepreneurial team are the “insiders” in the automotive industry. The team is based on close interpersonal relationships and has a high degree of cultural identity (Bae et al., 2014). There is no strict management assessment system for department management, which mainly depends on the affections of department leaders. All departments solve problems and formulate plans through discussion and negotiation, address problems with the least communication cost, accelerate product research and development, and make up for the impact of lack of workflow. Team members have played a great role in the entrepreneurship (Ireland et al., 2009), and cultural scenarios have become an important factor in the development of entrepreneurial enterprises.

### The institutional scenario can accurately predict and promote the enterprise performance

The entrepreneurial team can predict the development direction of policies and technical capabilities in advance, grasp and predict the development trend of new energy vehicles, and affect the performance of entrepreneurial enterprises. The Chinese government has provided a large amount of financing and information support for start-ups, guided business policies (O’Connor, 2013), supported entrepreneurial practices, macro-controlled the domestic market, and actively standardized the development of the industry. In the entrepreneurial process of Company A, under the institutional scenario of the entry mechanism maturing first, the exit mechanism maturing later, and the continuous optimization of the management mechanism, the entrepreneurial team fully considered and predicted the development trend of the new energy automobile industry policies. Meanwhile, they identified, evaluated, and took advantage of opportunities (Suddaby et al., 2015), took action to implement the response strategy in advance (Kuratko and Audretsch, 2009), and used the limited resources in the right direction of research and development, actively responded to possible policy changes and seized opportunities to achieve entrepreneurial performance.

### Economic scenarios negatively stimulate enterprise performance

The financial infrastructure in the economic scenario has a great impact on the performance of entrepreneurial enterprises. Financial infrastructure such as economic freedom (Mcmullen et al., 2008) and economic integration (Alhorr et al., 2010) affect the performance of entrepreneurial enterprises. The newly introduced investment organizations influence the political relationship of entrepreneurial enterprise performance and the continuation of entrepreneurial enterprises’ endogenous advantages, which is not conducive to entrepreneurial enterprise performance. The third stage is actually an unnecessary obstacle to entrepreneurship caused by financial facilities, which is a large negative factor in the current scenario and directly affects the entrepreneurial process in the third stage.

**Figure 3:**
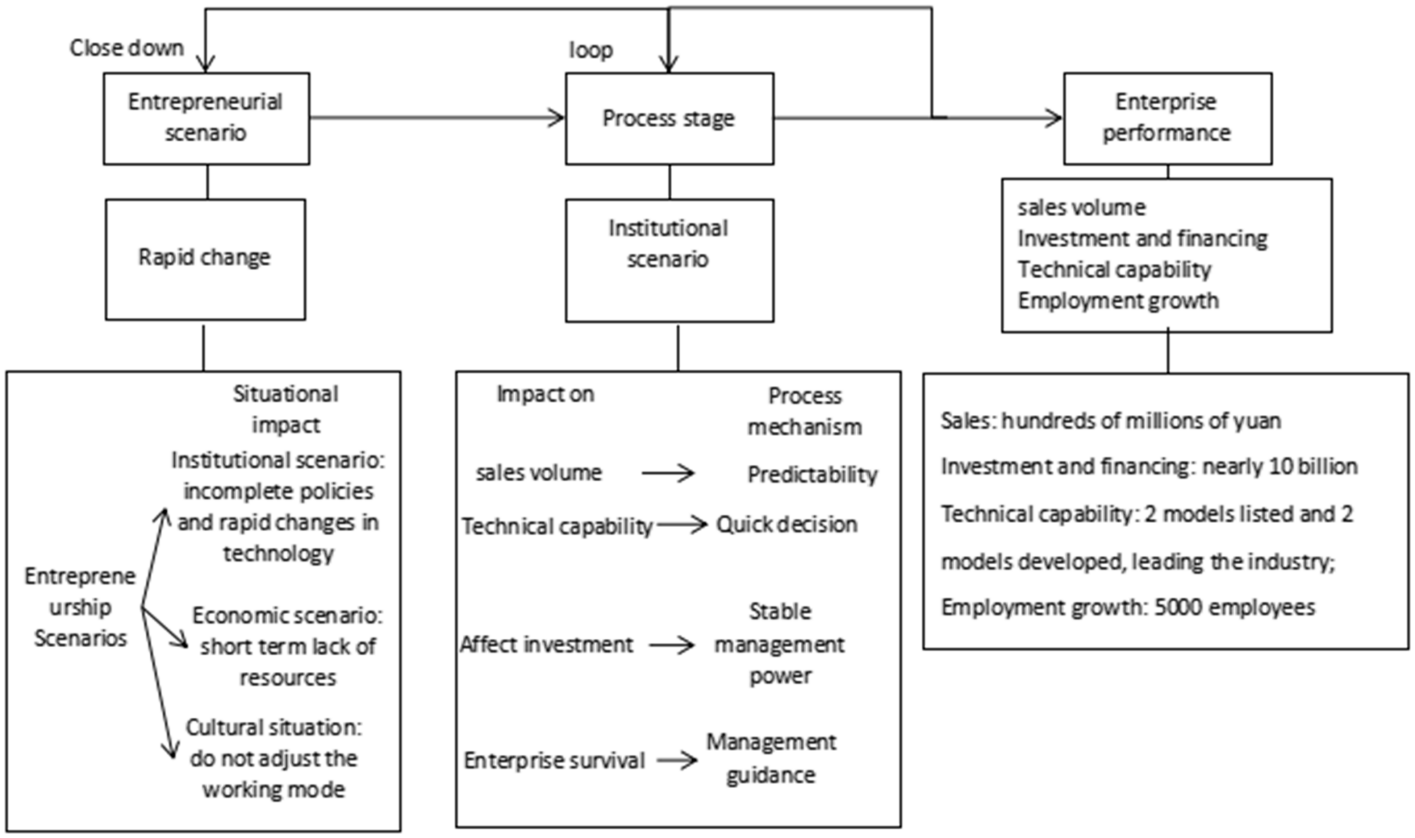
Scenario action process in rapidly changing context.

In the context of entrepreneurship in China, if entrepreneurial enterprises cannot quickly achieve business performance in the fierce market competition, they will soon go to failure. The conclusion of this paper focuses on the entrepreneurial scenario. According to the case analysis data, we sort out the functional mechanism of entrepreneurial scenario, as shown in Figure 3, which is the relationship between variables observed in this paper, and propose the realization process of enterprise performance under the entrepreneurial scenario in China.

It can be concluded from Figure 3 that in the context of entrepreneurship in China, the cultural context is the key scenario to achieve entrepreneurial enterprise performance. Of course, there are also some paradoxes in this study. For example, there is a conflict between the clear trend analysis of institutional scenarios and the fast decision-making hypothesis. Clear analysis requires sufficient time to investigate, understand and make decisions. However, in the case of rapid change, rapid decision-making can lead to rapid action to seize opportunities. This paradox also shows that for enterprises, it is more important for the management to be stable, understand the company’s current situation, understand the company’s future, and be familiar with the team atmosphere.

## Conclusion

The research case in this paper is based on the entrepreneurial scenario, which goes through the process of “human relationship management” to “system management.” The development process of entrepreneurial enterprises from personal care and trust management to institutional management is the process of survival and growth of entrepreneurial enterprises, as well as the embodiment of entrepreneurial enterprise performance. In the process from small micro entrepreneurial teams to large enterprise operation teams, the change process of cultural scenes is a typical embodiment of Chinese cultural scenes.

### Research theoretical contributions

This paper summarizes entrepreneurship theory from different perspectives, constantly verifies entrepreneurship practice and entrepreneurship theory, constantly revises entrepreneurship theory, constantly enriches entrepreneurship practice, and continuously explores entrepreneurship practice theory that conforms to Chinese situation through enterprise performance measurement in the process of entrepreneurship. It has a certain theoretical contribution to the process of entrepreneurship research, as shown below:

First of all, the existing scenario studies have studied the interaction between scenarios and entrepreneurial processes, the differences in entrepreneurial activities and the scarcity of resources in different contexts, but the research on China’s entrepreneurial context is insufficient. This paper enriches the experience summary and theoretical construction of entrepreneurial practice in China’s entrepreneurial context.

Secondly, in the existing research, most scholars prefer to measure financial performance indicators, while ignoring non-financial performance indicators (Tian and Ding, 2017). This paper enriches the evaluation and measurement theory of non-financial indicators and evaluates entrepreneurial performance through the analysis of non-financial performance indicators in product research and development and technical capabilities.

### Practical enlightenment of research

First, the matching of governance approaches and scenarios: innovation management and process management. The entrepreneurial team has rich experience, close relationship and strong will, which are crucial to the entrepreneurship and development of enterprises (Li and Li, 2009). This increases the sustainability and development possibility of the enterprise, strengthens the foundation for the enterprise to rapidly improve its technical capabilities, and ensures that the enterprise maintains rapid product development. The entrepreneurial team has had the work experience of perfect organizational structure, clear division of responsibilities and strict system, which has contributed to the strong work inertia of entrepreneurs. However, the management of entrepreneurial enterprises does not have a clear process definition. Compared with enterprises on the right track, team management is very different. Enterprise management needs to adopt different governance approaches at different stages of development to maximize team capacity, so as to maximize enterprise performance and growth (Brinckmann and Hoegl, 2011). In Phase 1, the main goal should be to better stimulate the overall efficiency of the team. With the gradual expansion of the enterprise scale and the gradual clarification of the division of labor, the enterprise process control should be strengthened to ensure the maximization of the overall work quality and efficiency of the enterprise.

Second, strategic choice and situation matching: follow-up strategy and leadership strategy. In the entrepreneurial context, entrepreneurial enterprises should flexibly match the necessary resources at different stages of development, and adopt different strategies to quickly match entrepreneurial scenarios. In the first stage, the “survival” of the enterprises is the biggest demand. There is almost no difference in information acquisition between different entrepreneurial enterprises, and it is difficult to predict the difficulty and risk of entrepreneurship. Therefore, at this stage, we should adopt a follow-up strategy, conform to the development trend, seize opportunities (Alvarez and Barney, 2010), and improve the “survival rate” of entrepreneurial enterprises. When enterprises have certain development capabilities, their positioning and prediction in the entrepreneurial field are more accurate. Different entrepreneurial enterprises have more and more differences in information prediction in terms of their own opportunities (Grégoire and Shepherd, 2012). With the development of business opportunities and resources, they should take the initiative to adopt innovative leadership strategies, seize development opportunities, and quickly increase the market share.

## Limitations and future prospects

Although this study provides a breakthrough theoretical and empirical summary for the study of entrepreneurial scenarios in China, the research results still have some limitations. This research is mainly based on the entrepreneurial enterprise research in China. In order to improve the universality of our research results, future research will use cases from different countries to test our research model.

Despite these limitations, existing studies have theoretically tested the mechanism between entrepreneurial scenarios and corporate performance. Next, we will further study the relationship between entrepreneurial scenarios and social performance on the basis of entrepreneurial orientation and corporate performance (Engelen et al., 2014).

## Data availability statement

The original contributions presented in the study are included in the article/supplementary material, further inquiries can be directed to the corresponding author.

## Ethics statement

Ethical review and approval was not required for the study on human participants in accordance with the local legislation and institutional requirements. Written informed consent from the patients/participants or patients/participants legal guardian/next of kin was not required to participate in this study in accordance with the national legislation and the institutional requirements.

## Author contributions

XL and JD: conceptualization and data curation. XL: formal analysis. HQ: funding acquisition, supervision, and project administration. XL and HQ: methodology. JD: resources and software. BZ: validation. XL: writing—original draft. XL, HQ, YX, and WW: writing—review and editing. All authors contributed to the article and approved the submitted version.

## Funding

This work was supported by National Natural Science Foundation of China (grant no. 71803180), and the Philosophy and Social Sciences Planning Project of the Ministry of Education in China (grant no. 18YJCZH140) and Development Fund of Zhejiang A&F University (2022FR008).

## Conflict of interest

The authors declare that the research was conducted in the absence of any commercial or financial relationships that could be construed as a potential conflict of interest.

## Publisher’s note

All claims expressed in this article are solely those of the authors and do not necessarily represent those of their affiliated organizations, or those of the publisher, the editors and the reviewers. Any product that may be evaluated in this article, or claim that may be made by its manufacturer, is not guaranteed or endorsed by the publisher.
